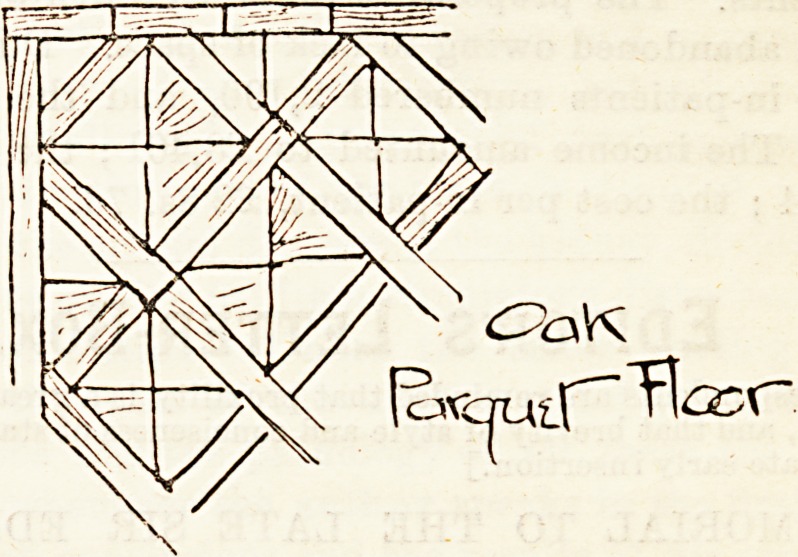# Floors for Hospital Wards

**Published:** 1895-06-15

**Authors:** 


### PRACTICAL DEPARTMENTS.

### FLOORS FOR HOSPITAL WARDS. (*Continued.*)

Recurring for a moment to the *construction* of floors for hospital wards, it must not be forgotten that it is almost as necessary for these to be sound-proof as fire-proof, and it is also desirable that they shall be so made as to allow of free inlets for air currents. Messrs. Homan and Rodgers, of Gracechurch Street, whose experience in this particular line has been varied and extensive, are, we find, of opinion that no perfectly solid floor can be said to be entirely sound-proof, and that an air space or chamber is essential to the attainment of this object. In several recently built hospitals the floors have been laid by this firm on this plan of construction, an air space of from four to six inches being given between floor and ceiling. The floors at the New Hospital for Women, Euston Road, the Evelina Hospital for Children, the new Parkwood Convalescent Home, Swanley, and many others have been laid by this firm, and in all the more recent instances on the air-space system. Messrs. Homan and Rodgers also contend for central heating of the wards, the floors being laid with a view to allow of fresh air inlets and smoke flues, each absolutely protected from the action of fire.

Where the floor surface is of wood, as is most usually the case in this country, the comparative advantages of scrubbed versus polished boards forms still a more or less vexed question amongst hospital authorities, inquiries failing to find anything like a consensus of opinion on this subject. In the older buildings scrubbed boards are generally the rule, but in modern hospitals polished oak or teak, or wood blocks, are now everywhere gaining ground. Our illustrations give an idea of each of these methods. No. 1 shows just the ordinary boarding, which should always be of oak or teak, the latter being the harder and in most respects the more satisfactory of the two woods. It is important to note that the boards should be quite narrow, and being carefully "tongued and grooved," that is, each plank fitting tightly into its neighbour, there can be no danger of dust and accompanying microbes filtering through. Objections are sometimes raised that highly polished floors are dangerous from their slipperiness, but if precautions are taken as at the Middlesex Hospital, and patients only allowed the list slippers provided in each ward, and crutches, &c., tipped with rubber, this difficulty vanishes promptly. Such floors, stained and well polished, are far superior to the form of flooring known as wood blocks, which is shown in illustration No. 2. This is preferred by some people because of its noiselessness (it is usually unpolished), and because scrubbing is credited with more virtue than properly belongs to it. It seems to stand to reason that moist wood can only make a very excellent culture ground for micro-organisms, while a polished surface is non-absorbent, and forms no such harbour of evil.

The third drawing illustrates what is known as a parquet flooring. This method is sometimes, very undesirably, made use of to avoid entire reflooring, the blocks being laid over the old original surface, thus simply covering out of sight the boards which have been impregnated with the dust and dirt of years. It cannot be too strongly affirmed that where reflooring is to be done at all it must be done thoroughly, or it will be worse than useless, and will lead to a far greater outlay in the end than if carried out as it should be in the first instance.

With regard to the treatment of wood floors, a method recommended by some authorities in the case of ordinary unstained boards is that of washing over twice or three times a week with Condy's fluid. This also makes a very satisfactory permanent staining, if well polished afterwards, though it usually requires renewing every now and then.

It is very essential, and is now being recognised by the best authorities, that there shall be no dust-collecting angles at the junction of wall and floor. The ceilings should be curved, corners rounded, and circular fillets inserted at the floor junction, to allow of absolute cleansing and washing of every corner. Mr. Burdett, in "Hospitals and Asylums of the World," says: "In all floors . . . the angle between the floors and the wall should be rounded to a radius of not less than two inches. Where the walls are of glazed brick this can be best effected by means of a specially made hollow-moulded brick. In wards where the floors are of wood, a hollowed fillet of oak best answers the purpose; and where a marble and cement floor joins a plastered or cemented wall, the hollow can best be formed in the material of which the floor is made."

To sum up, it may be said that terazzo or cement floors are the best from the sanitary and economic point of view. There can be no question of any harbouring of dirt; they lend themselves to easy cleansing and disinfecting. At the same time it must be said that well-laid polished teak or oak flooring is pleasanter to sight and touch, and where really well put down, is found quite satisfactory. Polishing involves really less labour than scrubbing, and from a sanitary aspect is certainly better. Scrubbed floors must therefore be relegated to the lowest place in the scale when considered in relation to their use in hospital wards.

## Figures and Tables

**Figure f1:**
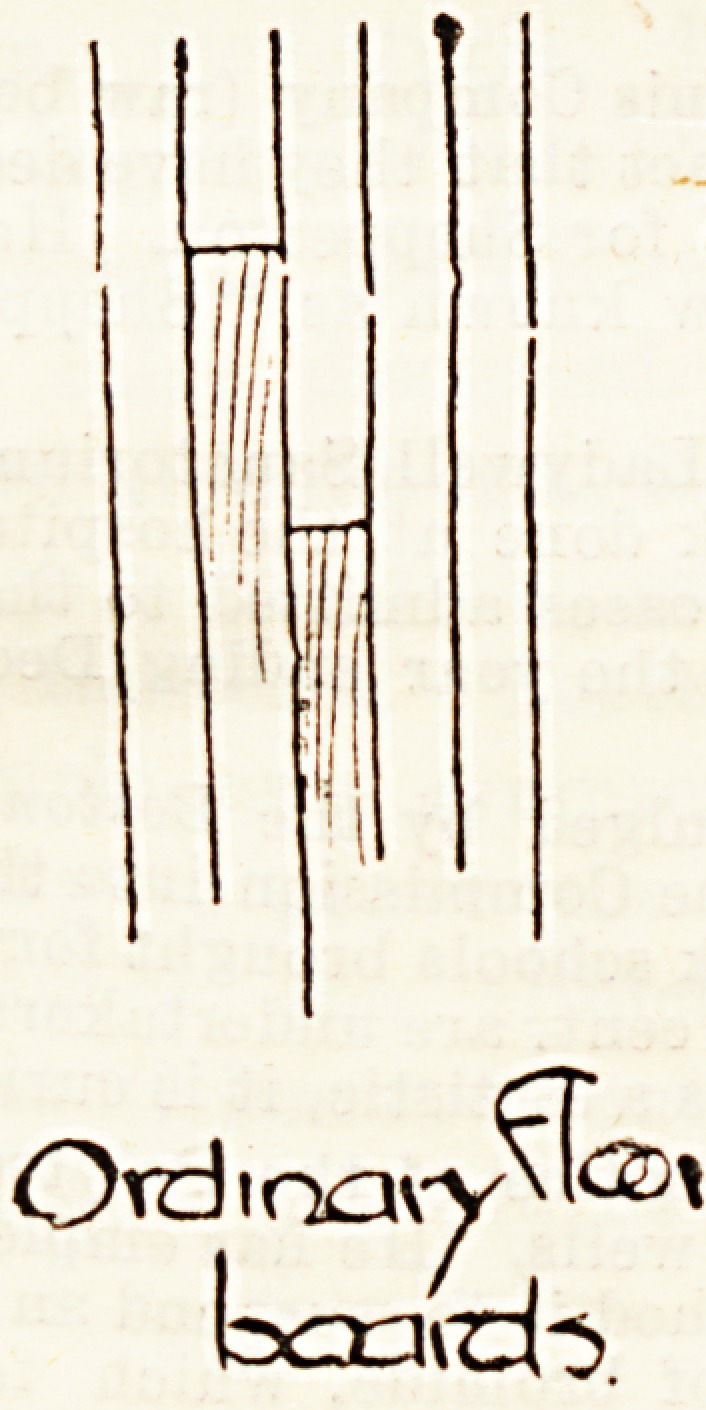


**Figure f2:**
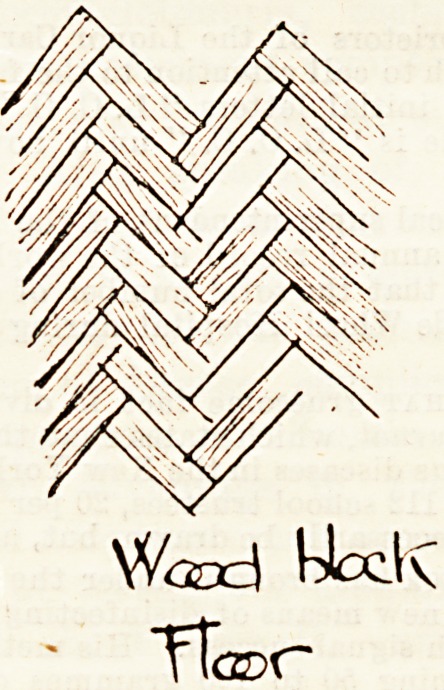


**Figure f3:**